# Misleading Imaging Findings: Bilateral Mylohyoid Defect Presenting as a Submandibular Mass Due to Sublingual Gland Protrusion

**DOI:** 10.3390/diagnostics14242833

**Published:** 2024-12-16

**Authors:** Dagnija Grabovska, Junsiyuan Li, Maija Radziņa, Arturs Balodis

**Affiliations:** 1 Institute of Diagnostic Radiology, Pauls Stradins Clinical University Hospital, LV-1002 Riga, Latviamaija.radzina@rsu.lv (M.R.); 2 Faculty of Residency, Latvian University, LV-1586 Riga, Latvia; 3 Department of Radiology, Sengkang General Hospital, Duke-NUS Medical School, Singapore 169857, Singapore; lijunsiyuan@gmail.com; 4 Department of Radiology, Riga Stradins University, LV-1007 Riga, Latvia

**Keywords:** sublingual gland, salivary gland diseases, muscles, magnetic resonance imaging

## Abstract

Background/Objectives: The muscular base of the oral cavity is formed of the mylohyoid muscle, which forms a sling inferior to the tongue. The muscle is often discontinuous, and defects may include salivary tissues, fat, and/or blood vessels. Hypertrophic sublingual glands located in mylohyoid defects can be herniated into bilateral submandibular spaces and present as palpable masses. The etiology of this condition may be congenital or acquired, and although such anatomical variations are common, they often go unrecognized in clinical practice. Sialoceles are cyst-like structures that result from chronic inflammation or ductal injury, indicating underlying problems with drainage efficiency. Methods: In this case series, we present two patients. Results: The first patient is a 44-year-old female who presented with a slowly enlarging right submandibular mass for two years, while the second is a 70-year-old female who presented with nonspecific neck discomfort, lacking palpable masses. In both, initial imaging (ultrasound and CT) was inconclusive. MRI revealed right sublingual gland herniation through a mylohyoid defect (mylohyoid boutonniere) in both cases. Conclusions: This highlights the importance of comprehensive imaging in the diagnosis of submandibular masses and emphasizes the need for considering mylohyoid boutonniere in cases of bilateral submandibular masses. Further research is warranted into the sialoceles associated with salivary gland abnormalities.

**Figure 1:**
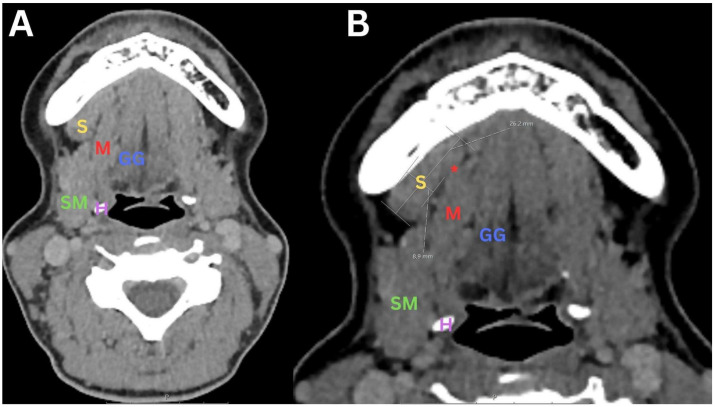
First patient: A 44-year-old female patient who experienced a slowly enlarging mass in the right submandibular region for two years; the mass was painless, mobile and firm, accompanied by no other significant complaints. Initial evaluation included an ultrasound examination, which revealed a mass in the right submandibular area; however, the precise diagnosis remained elusive, and the pathology was not correctly identified. Following an inconclusive ultrasound, a CT scan with intravenous contrast was performed. However, the underlying pathology remained unidentified, probably due to a lack of recognition of the mylohyoid muscle defect. (**A**) In the CT scan, an axial view shows a defect in the right mylohyoid muscle (M) located in its anterior third, which measures approximately 8–9 mm. There is a pronounced herniated sublingual salivary gland (S) with complete protrusion of the gland, which has caused the patient discomfort for the past two years. This condition manifested as a palpable mass and a sensation of discomfort. (**B**) This image provides an enhanced visualization of the location of the right mylohyoid defect (*) and the complete protrusion of the right sublingual salivary gland (S). In this image, the defect (*) in the right mylohyoid muscle (M) is more clearly delineated, allowing a better understanding of its anatomical context. The sublingual salivary gland (S) is prominently visible, fully protruding through the defect (*), which emphasizes the extent of the herniation. This clear visual representation helps to assess the condition and understand the relationship between the mylohyoid defect (*) and the protruding gland, which has contributed to the symptoms of the patient. S—sublingual gland; M—mylohyoid muscle; *—mylohyoid muscle defect; GG—genioglossus muscle; SM—submandibular gland; H—hyoid bone. Subsequently, the first patient underwent magnetic resonance imaging (MRI) of the soft tissues of the neck with intravenous contrast administration. The muscular base of the oral cavity is formed of the mylohyoid muscle, which forms a sling inferior to the tongue. It is inserted into the slightly obliquely oriented mylohyoid line on the middle surface of the mandible, with the posterior aspect more cranial than the anterior aspect. The muscle is thickest in the posterior region and thin when approaching the mental tubercle [1]. In fact, the muscle is often discontinuous. Anatomical and surgical literature has shown that defects may include sublingual or submandibular salivary tissues, fat, blood vessels, or all three components [2]. As described in the literature, hypertrophic sublingual glands located in mylohyoid defects can be herniated into bilateral submandibular spaces and present as palpable masses [2,3]. Most mylohyoid muscle defects are less than 5 mm, but may occasionally be greater than 2 cm. Consequently, larger herniations can be mistaken both clinically and radiologically for pathological abnormalities [4]. The etiology of this condition may be congenital or acquired, and although such anatomical variations are not uncommon and can be found in almost every fifth individual using ultrasound, they often go unrecognized in clinical practice [4]. Sialoceles are cyst-like structures that result from the obstruction of saliva drainage, often due to chronic inflammation or ductal injury. They are associated with conditions that restrict normal salivary function, indicating underlying problems with drainage efficiency [5]. In this case series, we present two patients. In both, the initial imaging (ultrasound and CT) was inconclusive. MRI revealed right sublingual gland herniation through a mylohyoid defect (mylohyoid boutonniere) in both cases. This highlights the importance of comprehensive imaging, especially MRI, in the diagnosis of submandibular masses to avoid unnecessary interventions and emphasizes the need for considering mylohyoid boutonniere in cases of bilateral submandibular masses. Further research is warranted into the management of sialoceles associated with salivary gland abnormalities.

**Figure 2:**
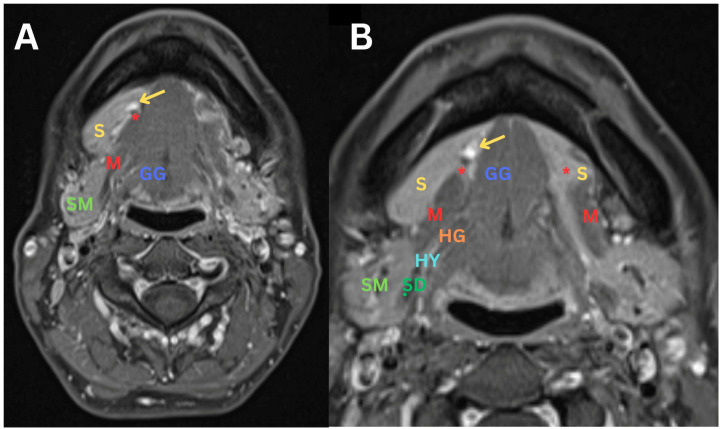
(**A**) An MRI T1 TSE Dixon axial sequence showing a defect in the right mylohyoid muscle (M), accompanied by the protrusion of the right sublingual salivary gland (S). (**B**) A closer view using the same T1 TSE Dixon axial sequence, highlighting the protrusion of the right sublingual salivary gland (S), with several small sialoceles indicated by an arrow in the background, suggesting a chronic process. Sialoceles occur in the context of mylohyoid boutonnieres due to the displacement of the sublingual gland through a defect or gap in the mylohyoid muscle. This anatomical variation leads to the accumulation of saliva in the surrounding tissue, as the herniated gland may become obstructed or damaged. The mylohyoid muscle typically supports the sublingual gland, and when a boutonniere defect is present, the muscle’s integrity is compromised, allowing the gland to prolapse and potentially disrupt normal salivary drainage. As a result, saliva can collect in the tissues, forming a sialocele. This condition highlights the importance of understanding the anatomical relationships and variations in the submandibular region to avoid misdiagnosis and inappropriate management. Similar but less pronounced changes are observed on the left side, where there is a minor protrusion of the left sublingual salivary gland (S). It is notable that the signal intensity of both sublingual salivary glands (S) is similar, and the contrast is homogeneous, with no MRI findings suggesting malignancy. S—sublingual gland; M—mylohyoid muscle; *—mylohyoid muscle defect; GG—genioglossus muscle; SM—submandibular gland; SD—submandibular duct (also known as Wharton’s duct); HG—hyoglossus muscle; HY—hypoglossal nerve.

**Figure 3:**
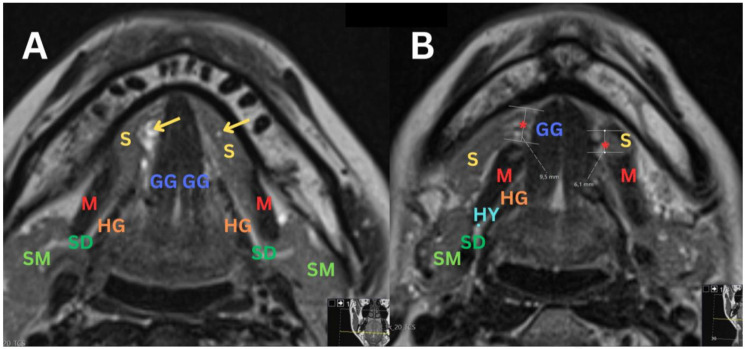
(**A**) An MRI of the soft tissues of the neck with intravenous contrast administration of T2 TSE Dixon axial sequence, showing a defect in the right mylohyoid muscle (M), accompanied by the protrusion of the right sublingual salivary gland (S) with several small sialoceles indicated by an arrow in the background. Similar but less pronounced changes are observed on the left side, where there is a minor protrusion of the left sublingual salivary gland (S) with small sialocele (**arrow**). (**B**) A closer view of the defects in the right mylohyoid muscle (M) using the same T2 TSE Dixon axial sequence, highlighting the protrusion of the right sublingual salivary gland (S). Similar but less pronounced changes are observed on the left side, where there is a minor protrusion of the left sublingual salivary gland (S). S—sublingual gland; M—mylohyoid muscle; *—mylohyoid muscle defect; GG—genioglossus muscle; SM—submandibular gland; SD—submandibular duct (also known as Wharton’s duct); HG—hyoglossus muscle; HY—hypoglossal nerve.

**Figure 4:**
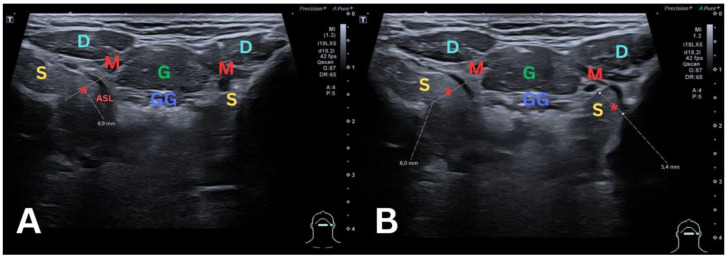
To clarify the changes, an ultrasound examination was performed. In the first image (**A**), the ultrasound examination, performed with a Canon Aplio i800 device, reveals a defect in the right mylohyoid muscle (M) measuring approximately 9 mm. This defect is associated with a protrusion of the right sublingual salivary gland (S), along with a protrusion of the right sublingual artery (ASL) through the muscle defect (*). In the second image (**B**), there is an observation of a defect (*) in the left mylohyoid muscle (M) measuring approximately 5 mm, accompanied by a small protrusion of the left sublingual salivary gland (S). These findings indicate significant anatomical changes in the sublingual region, highlighting the presence of muscle defects and glandular protrusions on both sides, which confirmed the diagnosis. S—sublingual gland; M—mylohyoid muscle; *—mylohyoid muscle defect; GG—genioglossus muscle; G—geniohyoid muscle; D—digastric muscle anterior belly; ASL—sublingual artery.

**Figure 5:**
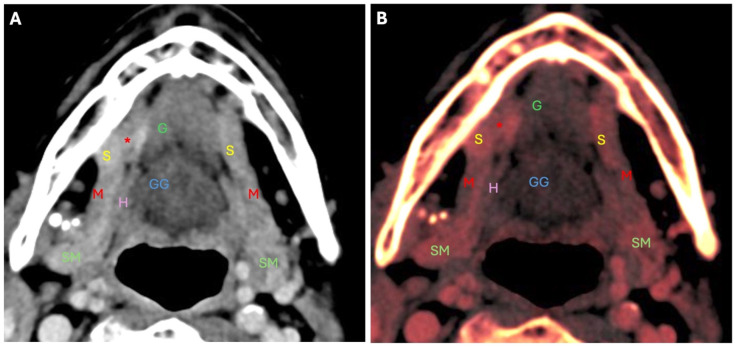
The second patient: dual-energy contrast-enhanced CT neck. A 70-year-old female who presented with nonspecific neck discomfort, lacking palpable masses. Initial imaging (ultrasound and CT) was inconclusive. MRI revealed a right sublingual gland herniation through a mylohyoid defect (mylohyoid boutonniere). In fact, this stable lesion had been present, unrecognized, for three years based on prior imaging. The axial CT image (**A**) shows a moderately enhancing nodular structure (S) in the right submandibular space, with a narrowed neck extending through a defect of the mylohyoid muscle (*) into the sublingual space. Note a partially imaged venous malformation with phleboliths in the right submandibular space, an incidental finding. An iodine map (**B**) discovered that the nodular structure has same degree of enhancement as the contralateral sublingual gland and both submandibular glands. S: sublingual gland; SM: submandibular gland; M: mylohyoid muscle; *: boutonniere; G: geniohyoid muscle; GG: genioglossus muscle; H: hyoglossus muscle.

**Figure 6:**
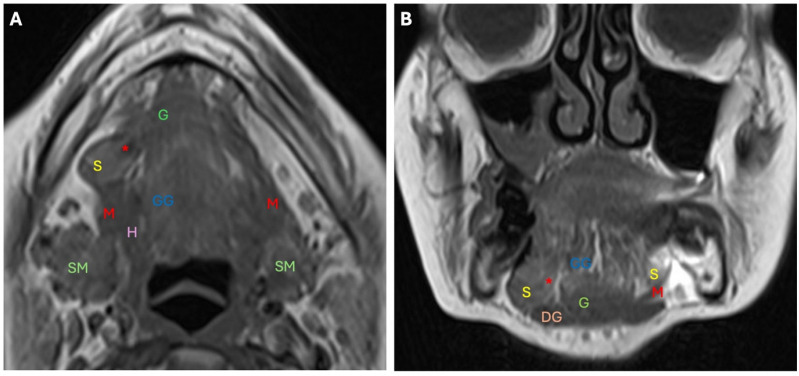
Axial (**A**) and coronal (**B**) T1-weighted images show a right mylohyoid boutonniere (*) through which the hypertrophic right sublingual gland (S) herniates into the right submandibular space. S: sublingual gland; SM: submandibular gland; M: mylohyoid muscle; *: boutonniere; G: geniohyoid muscle; GG: genioglossus muscle; H: hyoglossus muscle; DG: anterior belly of digastric muscle.

**Figure 7:**
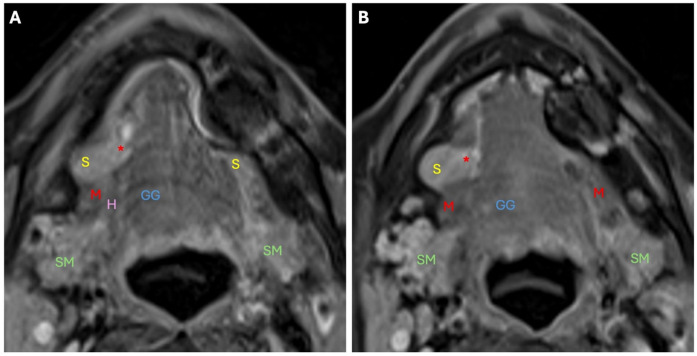
The two consecutive axial postcontrast T1-weighted fat-saturation images above (**A**) and below (**B**) show herniation of the right sublingual gland (S) through the right mylohyoid boutonniere (*) into the right submandibular space. The left sublingual gland is smaller in comparison. S: sublingual gland; SM: submandibular gland; M: mylohyoid muscle; *: Boutonniere; G: geniohyoid muscle; GG: genioglossus muscle; H: hyoglossus muscle.
